# Normal and Aberrant TALE-Class Homeobox Gene Activities in Pro-B-Cells and B-Cell Precursor Acute Lymphoblastic Leukemia

**DOI:** 10.3390/ijms231911874

**Published:** 2022-10-06

**Authors:** Stefan Nagel, Corinna Meyer

**Affiliations:** Department of Human and Animal Cell Lines, Leibniz-Institute DSMZ, German Collection of Microorganisms and Cell Cultures, 38124 Braunschweig, Germany

**Keywords:** E2F1, homeodomain, HOX-code, KLF1, NKL-code, TBX-code

## Abstract

Homeobox genes encode transcription factors regulating basic developmental processes. They are arranged according to sequence similarities of their conserved homeobox in 11 classes, including TALE. Recently, we have reported the so-called TALE-code. This gene signature describes physiological expression patterns of all active TALE-class homeobox genes in the course of hematopoiesis. The TALE-code allows the evaluation of deregulated TALE homeobox genes in leukemia/lymphoma. Here, we extended the TALE-code to include the stages of pro-B-cells and pre-B-cells in early B-cell development. Detailed analysis of the complete lineage of B-cell differentiation revealed expression of TALE homeobox genes IRX1 and MEIS1 exclusively in pro-B-cells. Furthermore, we identified aberrant expression of IRX2, IRX3 and MEIS1 in patients with B-cell precursor acute lymphoblastic leukemia (BCP-ALL) which originates from early B-cell progenitors. The data showed correlated activities of deregulated TALE-class members with particular BCP-ALL subtype markers, namely IRX2 with TCF3/E2A-fusions, IRX3 with ETV6/TEL-fusions, and MEIS1 with KMT2A/MLL-fusions. These correlations were also detected in BCP-ALL cell lines which served as experimental models. We performed siRNA-mediated knockdown experiments and reporter gene assays to analyze regulatory connections. The results showed mutual activation of IRX1 and TCF3. In contrast, IRX2 directly repressed wild-type TCF3 while the fusion gene TCF3::PBX1 lost the binding site for IRX2 and remained unaltered. IRX3 mutually activated fusion gene ETV6::RUNX1 while activating itself by aberrantly expressed transcription factor KLF15. Finally, KMT2A activated MEIS1 which in turn supported the expression of IRX3. In summary, we revealed normal TALE homeobox gene expression in early B-cell development and identified aberrant activities of IRX2, IRX3 and MEIS1 in particular subtypes of BCP-ALL. Thus, these TALE homeobox genes may serve as novel diagnostic markers and therapeutic targets.

## 1. Introduction

The development of lymphocytes, including B-cells, occurs mostly in the bone marrow and starts with the hematopoietic stem cell (HSC)-derived common lymphoid progenitor (CLP). Sequential early stages of developing B-cells comprise B-cell progenitors, pro-B-cells, pre-B-cells and finally naïve B-cells which migrate into lymph nodes to terminate their differentiation. B-cell precursor acute lymphoblastic leukemia (BCP-ALL) is a malignant disease derived from developing early B-cells in the bone marrow. BCP-ALL is the most frequent hematopoietic malignancy in children while being less common in adults [1]. The tumor cells can be classified according to chromosomal translocations, generating particular fusion genes [2,3]. However, specific gene mutations may serve as additional classifiers [4,5]. The most frequent chromosomal translocations and their corresponding fusion genes in BCP-ALL comprise t(12;21)(p13;q22) and ETV6::RUNX1, t(1;19)(q23;p13) and TCF3::PBX1, t(9;22)(q34;q11) and BCR::ABL1, and t(4;11)(q23;p13) and KMT2A::AFF1. These fusion genes are important drivers of the disease and serve as diagnostic markers to assign BCP-ALL subtypes and allocate patients to unique treatment protocols [1,6]. Most of them encode aberrant transcription factors (TFs) and chromatin modulators, highlighting transcriptional deregulation in the pathogenesis of BCP-ALL.

Hematopoietic differentiation processes are typically regulated at the transcriptional level [7,8,9]. Outstanding developmental TFs are encoded by homeobox genes which control basic decisions in cell and tissue differentiation. The homeodomains of these factors contain three helices which interact with DNA and cofactors to regulate target gene activities [10]. Sequence similarities of their conserved homeobox have been used to arrange these genes into 11 classes and several subclasses [11]. Antennapedia (ANTP) represents the largest class; it contains the 38 HOX genes in addition to the 48-member-strong NKL homeobox gene subclass. TALE-class homeobox genes represent evolutionarily ancient homeodomain factors and share a three amino acid residue loop extension (abbreviated as TALE) located between helix 1 and helix 2. The human genome contains 20 TALE homeobox genes including six IRX and three MEIS genes [12,13].

The human IRX genes are related to the homeobox gene iroquois from *Drosophila*. They are genomically arranged in two clusters, consisting of IRX1, IRX2 and IRX4 (located at chromosomal position 5p15) and of IRX3, IRX5 and IRX6 (16q12). The three MEIS genes are located apart at 2p14 (MEIS1), 15q14 (MEIS2) and 19q13 (MEIS3). IRX genes are active during embryogenesis and control the differentiation of particular tissues and organs [14]. IRX1 regulates the development of the limbs, gut, kidney and lung and plays an oncogenic role in several types of cancer [15]. IRX1 and IRX3 are aberrantly expressed in myeloid leukemia, demonstrating oncogenic activities in hematopoietic malignancies [16,17,18]. MEIS1 mediates the differentiation of various types of cells and tissues as well, including those of the hematopoietic compartment [19,20]. This gene is an important oncogenic target of aberrantly fused chromatin activator KMT2A in both lymphoid and myeloid leukemias [21,22].

Reflecting their physiological functions in development and differentiation, deregulated homeobox genes drive the generation of specific hematopoietic malignancies [23,24,25]. To evaluate the activities of major subgroups of homeobox genes in corresponding entities of hematopoietic tumors, we have generated the “NKL-code” and the “TALE-code” [25,26]. These gene signatures describe the physiological activities of respective NKL and TALE homeobox genes during hematopoiesis and assist in the identification of deregulated homeobox gene expression in leukemia/lymphoma patients. Using the myeloid TALE-code, we have recently detected physiological activity of TALE homeobox gene IRX1 exclusively in megakaryocyte erythroid progenitors (MEPs) during early myelopoiesis, and aberrant expression of IRX1, IRX3 and IRX5 in particular subtypes of acute myeloid leukemia (AML) [18]. Analysis of patient data and of IRX-positive AML model cell lines revealed the myeloid differentiation factors GATA1, GATA2 and KLF1 as regulators and targets of IRX factors in AML, thus creating physiological and aberrant gene regulatory networks [18].

Here, we have extended the reported lymphoid TALE-code to include the early B-cell stages pro-B-cells and pre-B-cells. These additional data served to identify physiological expression of IRX1 and MEIS1 in pro-B-cells and aberrant activity of IRX and MEIS genes in BCP-ALL patients and cell lines which were used to analyze regulatory relationships.

## 2. Results

### 2.1. TALE Homeobox Gene Activities in Early B-Cell Development

**Figure 1:**
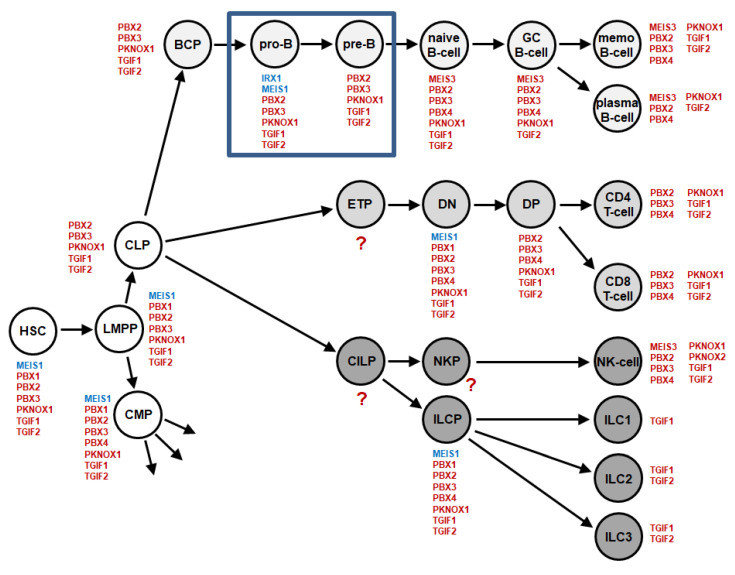
Extended lymphoid TALE-code. This diagram summarizes the screening results for expression analyses of TALE-class homeobox genes (red) in early hematopoiesis and lymphopoiesis. The newly analyzed stages for pro-B-cells and pre-B-cells are boxed in blue. We have termed this expression pattern lymphoid TALE-code. Expression of IRX1 and MEIS1 are highlighted in blue. Abbreviations: BCP, B-cell progenitor; CILP, common innate lymphoid progenitor; CLP, common lymphoid progenitor; CMP, common myeloid progenitor; DN, double negative; DP, double positive; ETP, early T-cell progenitor; GC, germinal center; HSC, hematopoietic stem cell; ILC, innate lymphoid cell; ILCP, innate lymphoid cell progenitor; LMPP, lymphomyelo-primed progenitor; macro, macrophage; memo B-cell, memory B-cell; NKP, NK-cell progenitor.

Recently, we have established the lymphoid TALE-code for early hematopoiesis and lymphopoiesis [26]. Here, we extend this gene signature to include two additional stages of early B-cell development, namely pro-B-cells and pre-B-cells, using dataset GSE19599 which covers selected hematopoietic progenitors [27]. The results are detailed in Figure S1 and depicted in Figure 1, showing the extended lymphoid TALE-code. Pro-B-cells expressed seven TALE homeobox genes including IRX1 and MEIS1 which were silenced in pre-B-cells. Thus, during B-cell development IRX1 and MEIS1 were exclusively expressed in this particular stage of B-cell differentiation. MEIS1 activity was also detected in hematopoietic stem cells and other lymphoid progenitors while IRX1 expression was restricted to pro-B-cells. According to RNA-seq gene expression data from the Human Protein Atlas, IRX and MEIS genes are generally silent or weakly expressed in hematopoietic cells and tissues, except for MEIS1 being predominantly expressed in progenitor cells. In contrast, they are highly active in other compartments (Figure S2). In the following, we examine the expression of IRX and MEIS genes in the context of B-cell malignancy.

### 2.2. Aberrant Expression of IRX2, IRX3 and MEIS1 in BCP-ALL Patients and Cell Lines

**Figure 2:**
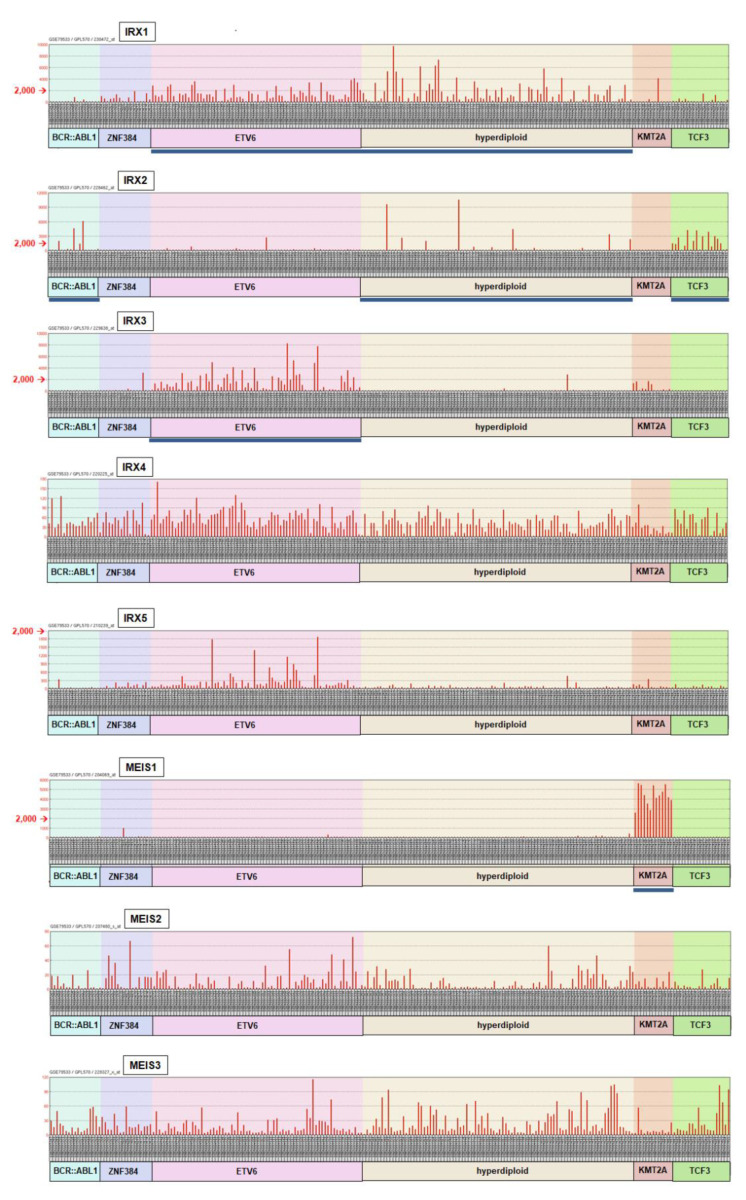
IRX and MEIS gene activities in BCP-ALL patients. Expression analyses of TALE homeobox genes IRX1, IRX2, IRX3, IRX4, IRX5, MEIS1, MEIS2 and MEIS3 in 226 pediatric BCP-ALL patients (dataset GSE79533). The patient samples are arranged according to the following indicated subtypes: BCR::ABL1, ZNF384, ETV6, hyperdiploid, KMT2A and TCF3. The expression level of 2,000 is highlighted. Subtypes containing at least two patients with significant expression levels (>2,000) are indicated by a bold blue line.

The early stages of B-cell development including pro-B-cell and pre-B-cell represent the cells of origin for BCP-ALL. Therefore, we wondered whether deregulated homeobox genes related to IRX1 and MEIS1 play an oncogenic role in this malignancy. To scrutinize the expression of IRX and MEIS genes in BCP-ALL patients, we screened the public expression profiling dataset GSE79533 which contains 226 pediatric patient samples [3]. The patients were arranged according to their assigned subtypes, containing the fusion gene BCR::ABL1, fusions with ZNF384, fusions with ETV6, hyperdiploid karyotypes, fusions with KMT2A and fusions with TCF3 (Figure 2). IRX1 showed the most consistent activity, while IRX4, IRX5, MEIS2 and MEIS3 were expressed at rather low levels below the significance limit. In contrast, significant expression of IRX2 was predominantly detected in BCP-ALL patients of the subtype TCF3 but also in the subtypes BCR::ABL1 and hyperdiploid. IRX3 showed significant activity in subtype ETV6, and MEIS1 in subtype KMT2A. Analysis of an additional dataset for BCP-ALL patients (GSE10792) confirmed widespread expression of IRX1 and restricted activities of IRX3 to subtypes ETV6 and KMT2A, and of MEIS1 to subtype KMT2A (Figure S3). However, this dataset lacks cases assigned to the subtypes ZNF384, hyperdiploid and TCF3. In summary, the physiologically expressed TALE homeobox gene IRX1 showed residual activity in most patient samples while IRX2, IRX3 and MEIS1 were aberrantly expressed in particular subtypes of BCP-ALL.

**Figure 3:**
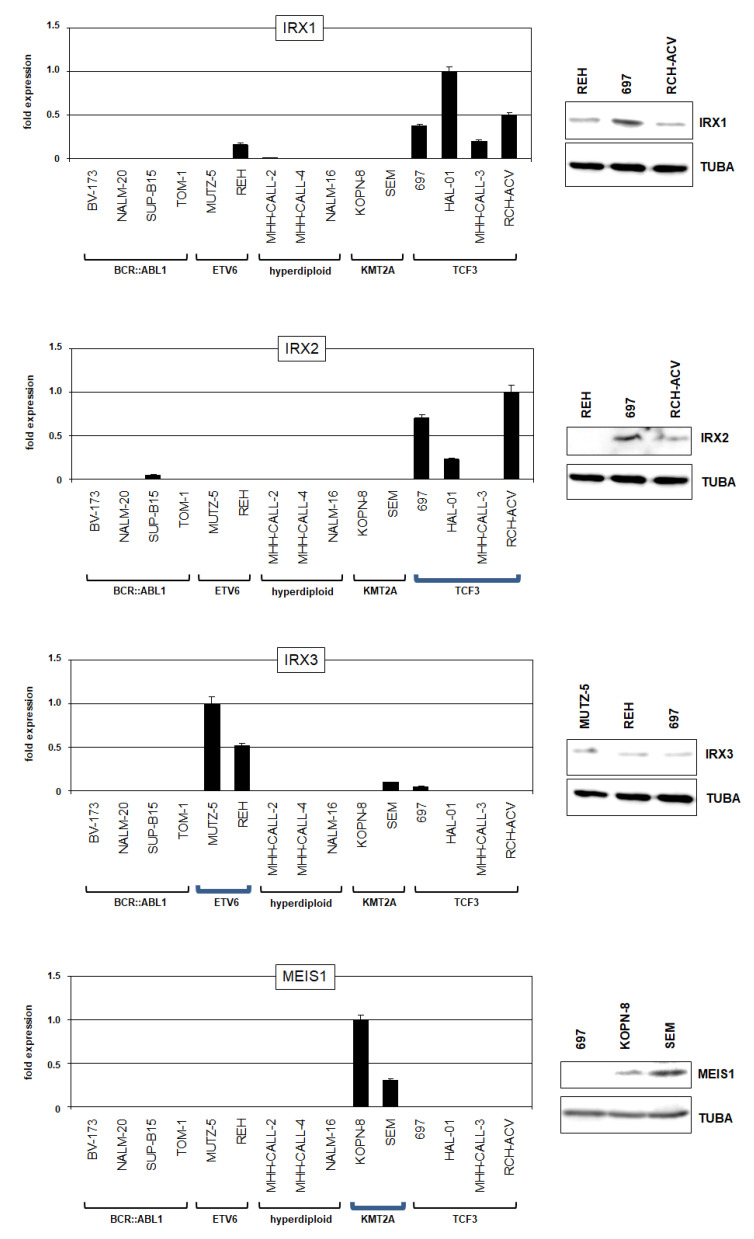
IRX and MEIS gene activities in BCP-ALL cell lines. Expression analyses of TALE homeobox genes IRX1, IRX2, IRX3 and MEIS1 in selected BCP-ALL cell lines as performed by RQ-PCR (**left**) and Western blot analyses (**right**). TUBA served as loading control. Cell lines were arranged according to reported subtypes as indicated below. Subtypes with corresponding gene activities detected in patients are highlighted by bold blue brackets.

To identify BCP-ALL cell line models for these deregulated TALE homeobox genes, we screened RNA-seq dataset LL-100 which contains 5 BCP-ALL cell lines (Figure S4) and performed RQ-PCR analysis of 15 BCP-ALL cell lines (Figure 3). The cell lines were grouped according to the subtypes BCR::ABL1, ETV6, hyperdiploid, KMT2A and TCF3. IRX1 expression was detected in cell lines of the subtypes ETV6 (REH) and TCF3 (697, HAL-01, MHH-CALL-3, RCH-ACV). IRX2 was weakly expressed in cell line SUP-B15 of subtype BCR::ABL1 and highly expressed in cell lines 697, HAL-01 and RCH-ACV of subtype TCF3. IRX3 was highly expressed in cell lines MUTZ-5 and REH (subtype ETV6) and weakly expressed in SEM (subtype KMT2A) and 697 (subtype TCF3). Finally, MEIS1 expression was detected just in cell lines KOPN-8 and SEM which belong to the subtype KMT2A. Western blot analysis showed expression of IRX1, IRX2, IRX3 and MEIS1 in selected BCP-ALL cell lines at the protein level (Figure 3), while RT-PCR analysis confirmed the presence of particular fusion genes (Figure S5), demonstrating that these cell lines are suitable models for examining the role of aberrantly expressed TALE homeobox genes in according BCP-ALL subtypes.

Of note, BCP-ALL cell line MUTZ-5 aberrantly expressed IRX3 but was negative for fusion gene ETV6::RUNX1 (Figure S5). RT-PCR analysis showed a rather complete absence of ETV6 expression exclusively in this cell line (Figure S5). Corresponding to these data, genomic profiling analysis showed the deletion of both ETV6 alleles in MUTZ-5 (Figure S6). This finding supports the conclusion that ETV6 represents a tumor suppressor gene in BCP-ALL and that ETV6 aberrations in general justify the assignment to the unique subtype ETV6.

In conclusion, our data for BCP-ALL patients and cell lines indicated predominant activity of aberrant IRX2 in subtype TCF3, of IRX3 in subtype ETV6, and of MEIS1 in subtype KMT2A, supporting a potential oncogenic role of these TALE-class homeobox genes in subsets of this malignancy. In the following, we thus examine mechanisms of deregulation and downstream effects of these genes.

### 2.3. E2F1 and TCF3 Are Regulators and Targets of IRX1 and IRX2 in BCP-ALL

Chromosomal rearrangements in malignant hematopoietic cells drive aberrant expression of oncogenes, including homeobox genes [23]. To analyze if this deregulating mechanism underlies activation of the identified TALE homeobox genes in BCP-ALL, we inspected the published karyotypes of the corresponding cell lines (www.DSMZ.de, accessed on 1 June 2022). In addition, we performed genomic profiling analysis of BCP-ALL cell lines 697, KOPN-8, MUTZ-5 and REH. Copy number data for chromosome 5 (containing the genes IRX1, IRX2 and IRX4), chromosome 16 (IRX3, IRX5, IRX6) and chromosome 2 (MEIS1) are shown in Figure S6. Except for REH, containing an additional chromosome 16 which may contribute to IRX3 expression, our examinations indicated the absence of targeted rearrangements at the loci of these TALE-class homeobox genes.

**Figure 4:**
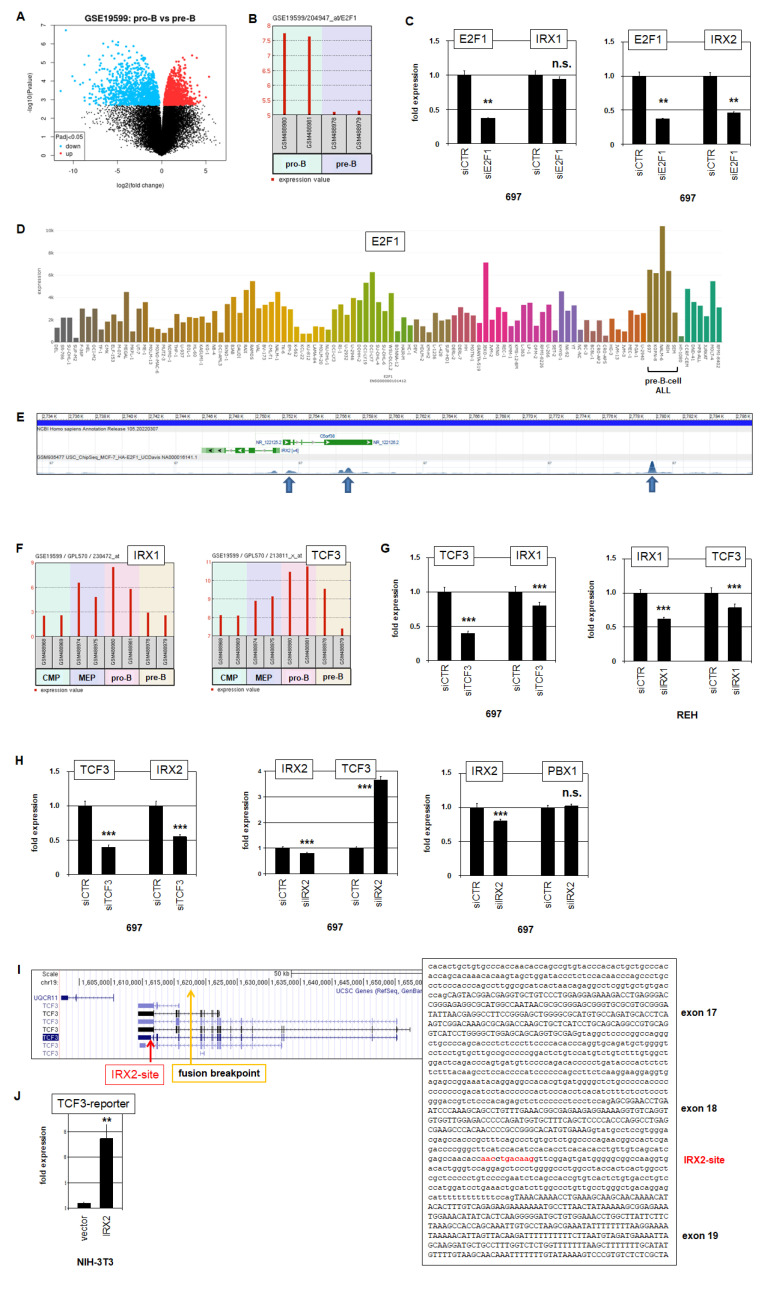
E2F1, TCF3, IRX1 and IRX2. (**A**) GEO2R analysis of expression profiling data from pro-B-cells and pre-B-cells (dataset GSE19599) was visualized as a volcano plot, showing significant differentially expressed genes in blue and red. (**B**) Bar plot showing expression data for E2F1 from pro-B-cells and pre-B-cells (dataset GSE19599). (**C**) RQ-PCR analysis of 697 cells treated for siRNA-mediated knockdown of E2F1. (**D**) RNA-seq data from 100 leukemia/lymphoma cell lines (LL-100) for E2F1 showing elevated expression levels in BCP-ALL-derived cell lines. (**E**) ChIP-seq data for E2F1 at the locus of IRX2 (obtained from ENCODE, dataset GSM935477). (**F**) Bar plot showing expression levels of IRX1 (left) and TCF3 (right) in hematopoietic progenitors (dataset GSE19599) including common myeloid progenitor (CMP), MEP, pro-B-cells and pre-B-cells. (**G**) RQ-PCR analysis of 697 cells treated for siRNA-mediated knockdown of TCF3 (left) and of REH cells treated for siRNA-mediated knockdown of IRX1 (right). (**H**) RQ-PCR analysis of 697 cells treated for siRNA-mediated knockdown of TCF3 (left) and IRX2 (middle and right). (**I**) Gene map and genomic sequence derived from the genome browser UCSC for the gene TCF3. A potential binding site for IRX2 is indicated in red (located in intron 18); the breakpoint for fusion gene TCF3::PBX1 is indicated in yellow (located in intron 16). (**J**) RQ-PCR analysis for reporter gene assay, using a genomic fragment from TCF3 intron 16, performed in NIH-3T3 cells. *p*-values are indicated by asterisks (** *p* < 0.01, *** *p* < 0.001, n.s. not significant).

To reveal potential regulators of IRX1, we compared expression profiling data of IRX1-positive pro-B-cells and IRX1-negative pre-B-cells using dataset GSE19599 and the associated online tool GEO2R which identified and illustrated differential gene activities (Figure 4A), and we generated a list of 250 genes showing the most statistically significant candidates. Accordingly, E2F1 was significantly elevated in pro-B-cells (*p* = 3.55 × 10^−^^5^) and encodes a TF; thus, it was chosen for detailed analysis (Figure 4B). We used BCP-ALL cell line 697 as a model and performed siRNA-mediated knockdown of E2F1. However, the expression level of IRX1 showed no alteration after this treatment (Figure 4C), indicating the absence of a regulatory input. In contrast, the expression level of IRX2 decreased (Figure 4C), demonstrating that E2F1 activated IRX2 in 697. RNA-seq data showed generally elevated E2F1 expression levels in BCP-ALL cell lines, including 697 (Figure 4D), which may reflect the physiologically enhanced activity of this gene in its cell of origin. Moreover, analysis of public ChIP-seq data (ENCODE project, dataset GSE935477) showed binding of E2F1 at the promoter and upstream region of IRX2 (Figure 4E), supporting direct activation. Thus, physiologically elevated expression of E2F1 in pro-B-cells underlies aberrant expression of IRX2 in BCP-ALL.

TCF3 encodes an important TF involved in lymphopoiesis, including B-cell development, operating as a tumor suppressor in this entity [28]. Analysis of gene expression profiling data of hematopoietic progenitors (dataset GSE19599) showed corresponding expression levels of IRX1 and TCF3 in MEPs and pro-B-cells, indicating a regulatory connection (Figure 4F). siRNA-mediated knockdown of TCF3 in 697 cells resulted in reduced expression of IRX1 (Figure 4G), indicating that TCF3 activated IRX1. siRNA-mediated knockdown of IRX1 in REH decreased expression of TCF3 (Figure 4G), demonstrating mutual activation of IRX1 and TCF3, and possibly reflecting the physiological situation in pro-B-cells.

TCF3 is targeted by chromosomal translocations creating fusion genes such as TCF3::PBX1 and TCF3::HLF in BCP-ALL [2,28], highlighting this gene in the context of IRX2 deregulation. siRNA-mediated knockdown of TCF3 in 697 cells also resulted in downregulation of IRX2 while knockdown of IRX2 indicated that IRX2 inhibited TCF3 expression (Figure 4H). Quantification of the expression levels of TCF3 fusion partner PBX1 in 697 cells showed no alteration after knockdown of IRX2 (Figure 4H), indicating that IRX2 regulated wild-type TCF3 but not fusion gene TCF3::PBX1. Searching for potential binding sites of IRX2 at the locus of TCF3 revealed a consensus site in intron 18 (Figure 4I). A reporter gene assay for this site demonstrated an activating input of IRX2 in NIH-3T3 cells (Figure 4J). Of note, although this assay showed an activating instead of an expected inhibitory effect, these data demonstrated direct interaction of IRX2 at intron 18 of TCF3. Interestingly, the breakpoint for fusion gene TCF3::PBX1 is located in intron 16 of TCF3. Thus, the fusion gene TCF3::PBX1 lacks the binding site for IRX2 and is, therefore, not a target for IRX2-mediated repression (Figure 4I). Taken together, these data revealed regulatory connections between IRX1, IRX2 and TCF3. IRX1 and TCF3 were physiologically coexpressed in pro-B-cells, probably operating as mutual activators, while aberrantly expressed IRX2 inhibited wild-type tumor suppressor gene TCF3 but not the oncogenic fusion TCF3::PBX1.

### 2.4. IRX3 Regulates ETV6 and KLF15 Regulates IRX3 in BCP-ALL

**Figure 5:**
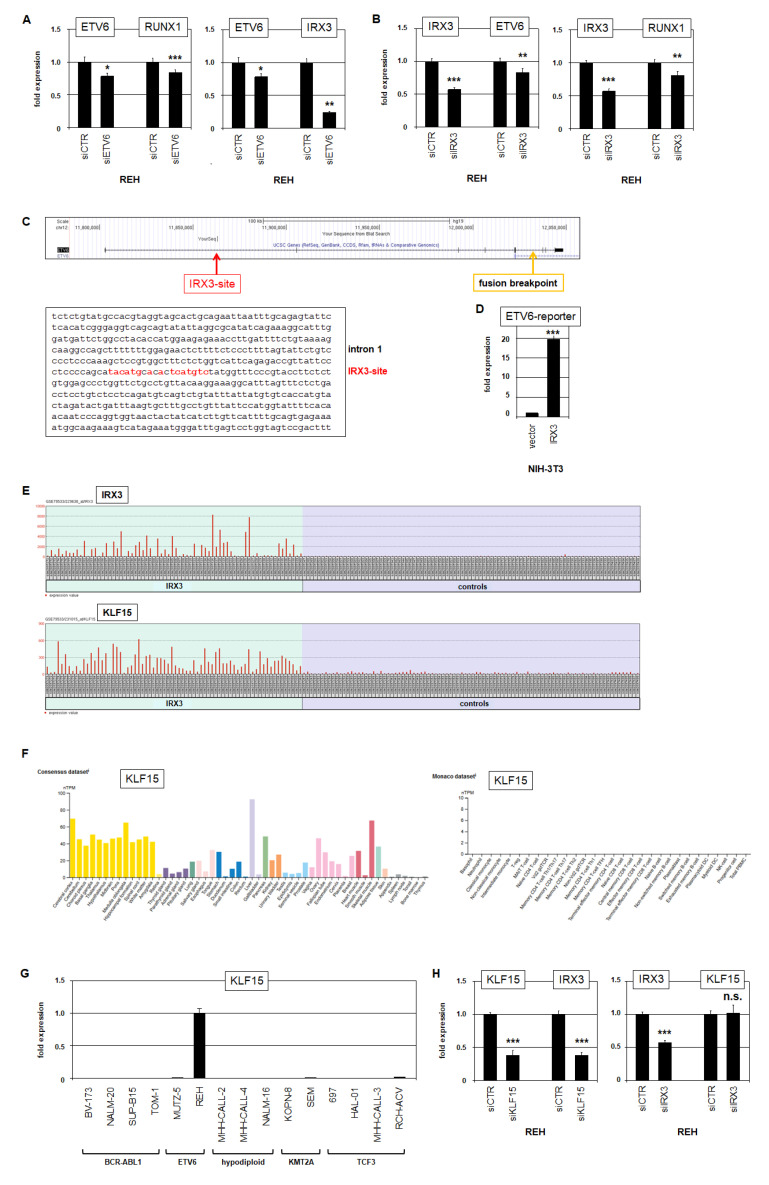
ETV6 and KLF15 are targets and regulators of IRX3. RQ-PCR analysis of REH cells treated for siRNA-mediated knockdown of (**A**) ETV6 and (**B**) IRX3. (**C**) Gene map and genomic sequence derived from the genome browser UCSC for the gene ETV6. A potential binding site for IRX3 is indicated in red (located in intron 1); the breakpoint for fusion gene ETV6::RUNX1 is indicated in yellow (located in intron 5). (**D**) RQ-PCR analysis for reporter gene assay, using a genomic fragment from ETV6 intron 1, performed in NIH-3T3 cells. (**E**) Expression profiling data for IRX3 and KLF15 from IRX3-positive and IRX3-negative control BCP-ALL patients using dataset GSE79533. (**F**) RNA-seq expression data for KLF15 from the Human Protein Atlas for various tissues (left) and hematopoietic cells (right). (**G**) RQ-PCR analysis of KLF15 in BCP-ALL cell lines. Subtypes of BCP-ALL are indicated. (**H**) RQ-PCR analysis of REH cells treated for siRNA-mediated knockdown of KLF15 and IRX3 (right). *p*-values are indicated by asterisks (* *p* < 0.05, ** *p* < 0.01, *** *p* < 0.001, n.s. not significant).

Aberrant expression of TALE homeobox gene IRX3 was predominantly detected in BCP-ALL patients and cell lines containing fusion gene ETV6::RUNX1 (Figure 2 and Figure 3). To analyze a potential regulatory connection, we performed siRNA-mediated knockdown experiments in REH cells that carry the ETV6::RUNX1 fusion. Knockdown of ETV6 resulted in concomitant downregulation of fusion partner RUNX1 and additionally of IRX3 (Figure 5A). Knockdown of IRX3 resulted in concomitant downregulation of ETV6 and RUNX1 (Figure 5B). These data indicated mutual activation of ETV6::RUNX1 and IRX3 in BCP-ALL. Searching for potential binding sites of IRX3 at the locus of ETV6 revealed a consensus site in intron 1 (Figure 5C). A reporter gene assay for this site demonstrated an activating input of IRX3 in NIH-3T3 cells (Figure 5C). Here, the binding site is present in both wild-type and translocated ETV6 genes. Of note, in REH cells, one allele of ETV6 was fused to RUNX1 while the other allele was deleted (Figures S4 and S5). Thus, IRX3 activated directly and exclusively the oncogenic fusion gene ETV6::RUNX1 in REH. MUTZ-5 cells expressed IRX3 but lacked ETV6::RUNX1. In this cell line, the loci for ETV6 were deleted (Figure S6), possibly to escape IRX3-mediated activation of tumor suppressor gene ETV6.

To identify additional regulators or target genes of aberrantly expressed IRX3 in BCP-ALL, we analyzed expression profiling data of patients using the public dataset GSE79533 and the associated online tool GEO2R. A comparison of IRX3-positive and IRX3-negative patients revealed the most significant 250 differentially expressed genes, including KLF15 (Figure 5E). Krüppel-like factors (KLFs) encode zinc-finger TFs involved in the transcriptional regulation of cell differentiation [29]. KLF1 encodes a master factor mediating hematopoietic differentiation of erythrocytes, while KLF15 is involved in the differentiation of kidney, liver and lung cells [29,30]. Accordingly, RNA-seq data analysis for KLF15 confirmed elevated expression levels in those tissues while showing absent activity in hematopoietic cells (Figure 5F). RQ-PCR analysis of BCP-ALL cell lines demonstrated KLF15 expression just in IRX3-positive cell line REH, corresponding to the patient data (Figure 5G). siRNA-mediated knockdown of KLF15 in REH resulted in reduced IRX3 expression, while knockdown of IRX3 did not alter KLF15 activity (Figure 5H). Thus, ectopically expressed KLF15 supported deregulated expression of IRX3 in BCP-ALL. Of note, analysis of the karyotype and genomic profiling data for REH showed the absence of rearrangements and copy number alterations at the locus of KLF15 (chromosomal position 3q21), excluding this mechanism for its aberrant activation (Figure S6).

### 2.5. MEIS1 Activates Expression of IRX3 in BCP-ALL

TALE-class homeobox gene MEIS1 is a well-known oncogene aberrantly activated via KMT2A-fusion proteins in both lymphoid and myeloid leukemias [21,22]. Our analyses showed physiological expression of MEIS1 in pro-B-cells and aberrant activity in the KMT2A subtype of BCP-ALL patients and cell lines KOPN-8 and SEM (Figure 2 and Figure 3). Furthermore, MEIS1-positive patients and cell line SEM coexpressed IRX3 at a lower level, indicating a regulatory connection. We have recently shown that HOXA10 mediates aberrant activation of IRX3 in myelomonocytic AML [18]. Considering that HOXA genes are activated by KMT2A and/or MEIS1, we speculated about a regulatory connection between these three genes in BCP-ALL subsets [21,31].

**Figure 6:**
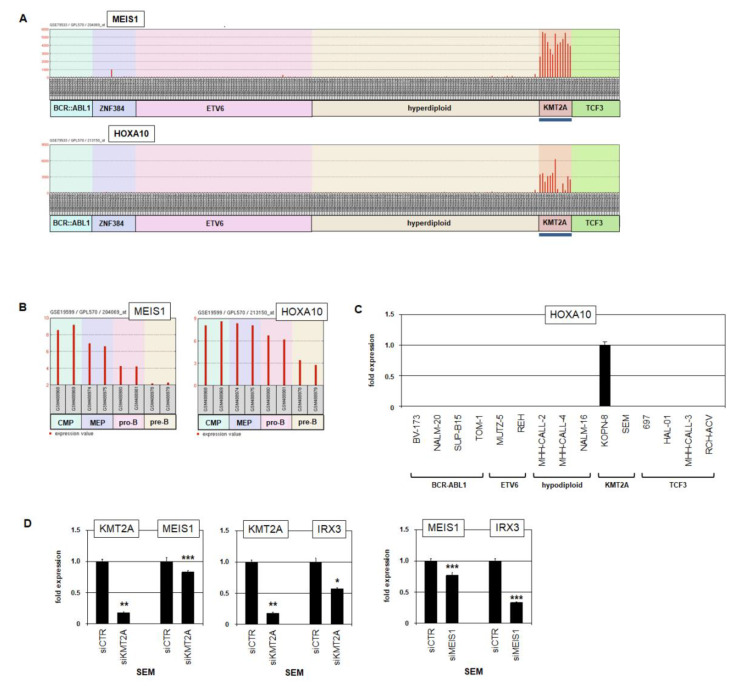
MEIS1 activates IRX3. (**A**) Expression profiling data for MEIS1 and HOXA10 from BCP-ALL patients using dataset GSE79533. Subtypes showing significant expression levels are indicated by a blue line. (**B**) Expression profiling data for MEIS1 and HOXA10 from hematopoietic progenitor cells including CMP, MEP, pro-B-cells and pre-B-cells using dataset GSE19599. (**C**) RQ-PCR analysis of HOXA10 in BCP-ALL cell lines. Subtypes of BCP-ALL are indicated. (**D**) RQ-PCR analysis of SEM cells treated for siRNA-mediated knockdown of KMT2A (left, middle) and MEIS1 (right). *p*-values are indicated by asterisks (* *p* < 0.05, ** *p* < 0.01, *** *p* < 0.001, n.s. not significant).

Analysis of MEIS1 and HOXA10 expression in BCP-ALL patients (Figure 6A) and selected hematopoietic progenitors (Figure 6B) demonstrated a strong correlation, supporting the indicated potential regulatory interactions. However, RQ-PCR analysis of BCP-ALL cell lines detected HOXA10 expression in KOPN-8 but not in SEM, thus contradicting the potential roles of aberrant KMT2A and/or MEIS1 in HOXA10 activation (Figure 6C). siRNA-mediated knockdown of KMT2A in HOXA10-negative SEM resulted in reduced expression of both MEIS1 and IRX3 (Figure 6D). Furthermore, knockdown of MEIS1 in SEM resulted in reduced expression of IRX3 as well (Figure 6D), demonstrating that MEIS1 activated IRX3. Thus, our data indicated that coexpression of MEIS1 and IRX3 in BCP-ALL subtype KMT2A reflects activation of MEIS1 by KMT2A-fusions and of IRX3 by MEIS1. HOXA10 is probably not involved in IRX3 deregulation in BCP-ALL.

### 2.6. Functional Analysis of IRX2 and IRX3 in BCP-ALL

TALE-class homeobox genes encode TFs involved in the regulation of basic developmental processes [12]. Aberrant activity of these factors in tumor cells may perturb their differentiation and thus contribute to carcinogenesis. However, deregulated homeobox genes influence additional oncogenic processes as well. Therefore, we analyzed the impact of IRX2 and IRX3 on proliferation and apoptosis of BCP-ALL cells by live-cell imaging.

**Figure 7:**
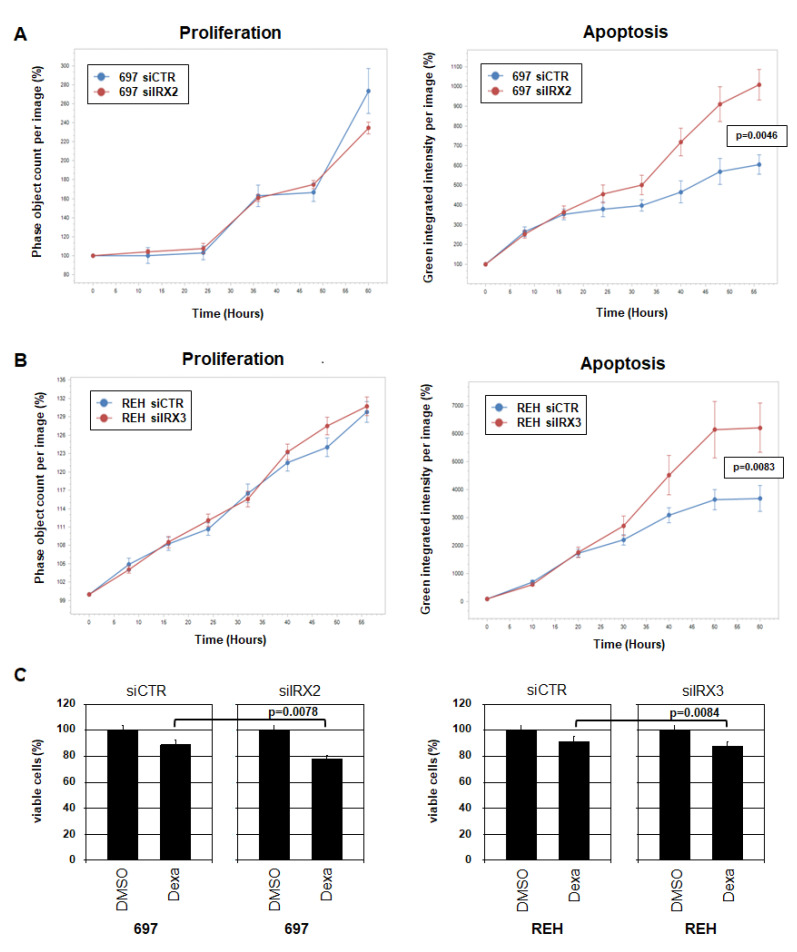
Oncogenic activities of IRX2 and IRX3 in BCP-ALL. (**A**) Live-cell imaging analysis of 697 cells treated for siRNA-mediated knockdown of IRX2. (**B**) Live-cell imaging analysis of REH cells treated for siRNA-mediated knockdown of IRX3. Standard deviations are shown as bars. Indicated *p*-values refer to terminal time points of treated versus control cells. (**C**) MTT assay analysis of 697 (left) and REH (right) treated for IRX knockdown and with 1 µM dexamethasone (Dexa) for 24 h, showing the percentage of viable cells. Standard deviations and *p*-values are indicated (*t*-test).

BCP-ALL cell line 697 was treated for siRNA-mediated knockdown of IRX2 and monitored for 60 h. While no effects on proliferation could be detected, the data showed an inhibitory role in apoptosis (Figure 7A). BCP-ALL cell line REH was accordingly monitored after knockdown of IRX3. The data demonstrated a similar picture: IRX3 did not impact proliferation but inhibited apoptosis (Figure 7B). These experiments indicated that deregulated IRX genes operate anti-apoptotically in BCP-ALL.

Dexamethasone is a chemotherapeutic drug used to treat hematopoietic malignancies, including BCP-ALL [32]. Treatment of 697 and REH cells for knockdown of the corresponding IRX genes and additionally with dexamethasone resulted in weak but significantly increased apoptosis as analyzed by MTT assay after 24 h (Figure 7C). These data support the anti-apoptotic role of aberrantly expressed IRX2 and IRX3 in BCP-ALL and highlight these IRX oncogenes as novel therapeutic targets in combination with conventional chemotherapeutic drugs. Taken together, the results of this study showed that aberrantly expressed IRX genes perform oncogenic functions in BCP-ALL via (i) deregulation of developmental TFs mediating the disturbance of B-cell differentiation and (ii) inhibition of apoptosis.

## 3. Discussion

In this study, we completed the lymphoid TALE-code, showing TALE homeobox gene activities in normal lymphopoiesis [26]. Previously, we described the NKL-code and TBX-code, which represent respective gene signatures of NKL homeobox genes and T-box genes in lymphopoiesis [25,33]. These codes show the physiological activities of selected TF-encoding gene groups in hematopoietic entities and allow the evaluation of TF activities in corresponding malignancies of patients. Here, analysis of the extended TALE-code data for stages of developing B-cells revealed restricted expression of IRX1 and MEIS1 in pro-B-cells. Subsequent analysis of corresponding BCP-ALL patient data demonstrated aberrant expression of three TALE-class members especially in particular subtypes of BCP-ALL: IRX2 in subtype TCF3, IRX3 in subtype ETV6, and MEIS1 in subtype KMT2A. Identified cell line models were used for functional analyses revealing subtype-specific activities.

IRX1 is physiologically expressed in pro-B-cells during lymphopoiesis and in MEPs during myelopoiesis [18]. Both cell types represent progenitors, but in contrast to pro-B-cells which generate exclusively B-cells, MEPs are able to differentiate into diverse hematopoietic cell types, namely megakaryocytes or erythrocytes. Pro-B-cells and MEPs express in addition to IRX1 other developmental TFs which were shown to regulate each other. In MEPs, GATA1 and GATA2 activate IRX1 while KLF1 and TAL1 are target genes of IRX1 [18]. For pro-B-cells, our data indicated that IRX1 and TCF3 were mutual activators. TCF3 encodes two isoforms of a helix–loop–helix TF family member and orchestrates B-cell differentiation together with EBF1, FOXO1 and PAX5 [34,35,36]. Thus, our data showed that IRX1 represents a novel player in the transcriptional regulation of early B-cell development.

In BCP-ALL, TCF3 and ETV6 in addition to other TF-encoding genes are rearranged via chromosomal translocations, disturbing the function of these TFs in early B-cell differentiation [34,37]. The generated fusion genes are both oncogenic drivers and markers for establishing subtypes of BCP-ALL [1,2,3,4,5,6]. Our data revealed interesting regulatory connections between aberrantly expressed IRX genes and particular fusion genes. IRX2 repressed wild-type TCF3 via a binding site located in intron 18. TCF3-fusion genes have lost this site and were, therefore, not targets of repressor IRX2. Furthermore, IRX3 activated ETV6 via a binding site located in intron 1 which is present in both wild-type ETV6 and ETV6-fusion genes. Deletion of the wild-type allele of ETV6 in REH cells supported the tumor suppressor status of this gene and indicated a potential mechanism of escaping IRX3-mediated activation. Cell line MUTZ-5 contains the uncharacterized chromosomal aberration t(12;13)(p12;q13-14) which may be etiologically related to the deletion of both alleles of ETV6 at 12p13 [38]. Accordingly, ETV6 deletion prevents activation by IRX3 in this cell line as well. Taken together, these regulatory relationships may underlie our observed correlation of IRX2 and IRX3 expression levels to the corresponding subtypes of BCP-ALL.

TALE homeobox gene MEIS1 showed physiological activity in several progenitors, including pro-B-cells, and is a reported target gene of rearranged KMT2A [21,22]. Accordingly, we detected aberrant MEIS1 expression in BCP-ALL subtype KMT2A. Coexpression of MEIS1 and IRX3 in this subtype may underlie the identified activation of IRX3 by MEIS1. However, this relationship was not significant in the patient data.

We identified E2F1 and KLF15 as transcriptional activators of aberrantly expressed IRX genes in BCP-ALL. E2F1 represents a ubiquitously expressed activatory TF, regulating basic cellular functions such as proliferation and apoptosis [39]. Pro-B-cells physiologically expressed elevated levels of E2F1 which mediated direct activation of aberrant IRX2 expression. In pro-B-cell line BaF-B03, E2F1 has been shown to support proliferation and survival if overexpressed [40]. Accordingly, we observed survival effects of E2F1 target gene IRX2 in 697 cells. KLF15 is a member of the KLF family which contains several tissue-specific TFs [29]. We showed that ectopically expressed KLF15 activated IRX3 in BCP-ALL cells. Normally, KLF15 is involved in the differentiation of kidney, liver and lung cells [29]. KLF family member KLF1 is hematopoietically expressed and involved in the differentiation of erythrocytes [30]. In MEPs, KLF1 represents a physiological target gene of IRX1 [18]. Thus, these data highlight physiological and oncogenic regulatory relationships between IRX and KLF TFs.

In conclusion, our study revealed the functional roles of TALE factors in normal and aberrant B-cell lymphopoiesis. IRX1 is a novel physiological TF active in B-cell development and part of a gene regulatory network controlling early B-cell differentiation. Aberrantly activated TALE homeobox genes IRX2, IRX3 and MEIS1 may disturb or deregulate developmental processes in B-cell development, driving the generation of BCP-ALL subtypes. Thus, our study contributes to the understanding of normal and abnormal processes in lymphopoiesis. Finally, deregulated TALE homeobox genes in BCP-ALL may serve as novel markers and potential therapeutic targets.

## 4. Materials and Methods

### 4.1. Analysis of Expression Profiling and RNA-Seq Data

Expression data for normal cell types were obtained from Gene Expression Omnibus (GEO, www.ncbi.nlm.nih.gov, accessed on 1 June 2022), using the expression profiling dataset GSE19599 [27], in addition to RNA-seq data from the Human Protein Atlas (www.proteinatlas.org, accessed on 1 June 2022) [41]. For analysis of cell lines, we exploited RNA-seq data from 100 leukemia/lymphoma cell lines (termed LL-100), available at ArrayExpress (www.ebi.ac.uk/arrayexpress, accessed on 1 June 2022) via E-MTAB-7721 [42]. Gene expression profiling data from BCP-ALL patients were examined using datasets GSE79533 and GSE10792 [3,43]. The associated online tool GEO2R was used to compare two selected groups, revealing the top 250 significant differentially expressed genes [44]. Of note, expression profiling datasets have been performed on chips lacking the TALE homeobox genes IRX6 and TGIF2LX.

### 4.2. Cell Lines and Treatments

Cell lines used in this study are held at the DSMZ (Braunschweig, Germany). Information concerning cultivation, classification and karyotype is given on the website of the cell bank (www.DSMZ.de, accessed on 1 June 2022). All cell lines had been authenticated and tested negative for mycoplasma infection. Gene-specific siRNA oligonucleotides were used to modify gene expression levels with reference to AllStars Negative Control siRNA (siCTR) obtained from Qiagen (Hilden, Germany). siRNAs (80 pmol) were transfected into 1 × 10^6^ cells by electroporation using the EPI-2500 impulse generator (Fischer, Heidelberg, Germany) at 350 V for 10 ms. After 20 h cultivation, electroporated cells were harvested.

The IncuCyte S3 Live-Cell Analysis System including the software module Cell-By-Cell (Essen Bioscience, Hertfordshire, UK) was used for functional tests of treated cells. Apoptotic cells were detected using the IncuCyte Caspase-3/7 Green Apoptosis Assay diluted at 1:2.000 (Essen Bioscience). Live-cell imaging experiments were performed twice with fourfold parallel tests. For the MTT assay, cell lines were transfected as indicated and treated for 20 h with 1 µM dexamethasone (Sigma, Taufkirchen, Germany) dissolved in dimethylsulfoxide (DMSO). Treated cells were prepared for standardized MTT (3-(4,5-dimethylthiazol-2-yl)-2,5-diphenyltetrazolium bromide; obtained from Sigma) assays. The measurement was performed twice in triplicates. The absorbance was determined at 405 nm and at 620 nm as background control using ELISA reader Multiskan EX (Thermo Electron, Vantaa, Finland).

### 4.3. Polymerase Chain Reaction (PCR) Analyses

TRIzol reagent (Invitrogen, Darmstadt, Germany) was used to extract total RNA from cultivated cells. cDNA was synthesized using 1 µg RNA, random priming and Superscript II (Invitrogen). Real-time quantitative (RQ) PCR analysis was performed using the 7500 Real-time System and commercial buffer and primer sets (Applied Biosystems/Life Technologies, Darmstadt, Germany). For normalization of expression levels, we quantified the RNA transcripts of the TBP gene. Quantitative analyses were performed as biological replicates and measured in triplicate. Standard deviations are presented in the figures as error bars. Statistical significance was assessed by *t*-test (two-tailed), and the calculated *p*-values are indicated by asterisks (* *p* < 0.05, ** *p* < 0.01, *** *p* < 0.001, n.s. not significant).

For the detection of fusion transcripts, we performed reverse transcription (RT) PCR, using oligonucleotides as reported previously [45]. In addition, we analyzed ETV6 and YY1, using the following oligonucleotides: ETV6-for 5′-AGGCCAATTGACAGCAACAC-3′, ETV6-rev 5′-TGCACATTATCCACGGATGG-3′, YY1-for 5′-AAGCAGGTGCAGATCAAGAC-3′, YY1-rev 5′-CCGAGTTATCCCTGAACATC-3′. PCR products were generated using taqpol (Qiagen) and thermocycler TGradient (Biometra, Göttingen, Germany), analyzed by gel electrophoresis and documented with the Azure c200 Gel Imaging System (Azure Biosystems, Dublin, CA, USA). All oligonucleotides were obtained from Eurofins MWG (Ebersberg, Germany).

### 4.4. Reporter Gene Assays

Sequences for consensus binding sites of IRX2 and IRX3 were obtained from the CIS-BP database (http://cisbp.ccbr.utoronto.ca/index.php, accessed on 1 June 2022) [46]. For the creation of reporter gene constructs, we amplified a reporter gene (HOXA9) and regulatory genomic fragments which were derived from intronic regions of TCF3 or ETV6 via PCR. We cloned the regulator fragments and the reporter gene (comprising exon1–intron1–exon2 of HOXA9), into the HindIII/BamHI and EcoRI sites, respectively, of the expression vector pcDNA3 downstream of the CMV enhancer as reported previously [47]. The oligonucleotides used for the amplification of the regulators were obtained from Eurofins MWG. Their sequences were as follows: TCF3-for 5′-ACAAGCTTTCAGCCCTGTGCTCTGGCCCCAG-3′, TCF3-rev 5′-CAGGATCCATGGGACAGGTCACAGAGTGAC-3′, ETV6-for 5′-GAAAGCTTGATTTTCTGTAAAAGCAAGGCCAGC-3′, ETV6-rev 5′-TAGGATCCTCTCAATATCACAACCTTTTGCCTTC-3′. Introduced restriction sites used for cloning are underlined. Constructs were validated by sequence analysis (Eurofins MWG). Transfections of plasmid DNA into NIH-3T3 cells (DSMZ) were performed using SuperFect Transfection Reagent (Qiagen). Commercial HOXA9 and TBP TaqMan assays (Thermo Fisher Scientific) were used for RQ-PCR analysis to quantify the spliced reporter-transcript, corresponding to the regulator activity.

### 4.5. Protein Analysis

Protein lysates from cell lines were prepared using SIGMAFast protease inhibitor cocktail (Sigma). Proteins were transferred onto nitrocellulose membranes (Bio-Rad, München, Germany) by the semi-dry method and blocked with 5% dry milk powder dissolved in phosphate-buffered saline buffer (PBS). We used the following antibodies: alpha-tubulin (Sigma, #T6199), IRX1 (Biozol, Eching, Germany, #DF3225), IRX2 (Biozol, #LS-C800571), IRX3 (Biozol, #MBS8223417), MEIS1 (Abcam, Cambridge, UK, #ab19867). For loading control, blots were reversibly stained with Ponceau (Sigma, Taufkirchen, Germany). In addition, we quantified alpha-Tubulin (TUBA). Secondary antibodies were linked to peroxidase for detection by Western Lightning ECL (Perkin Elmer, Waltham, MA, USA). We used the digital system ChemoStar Imager (INTAS, Göttingen, Germany) for documentation.

### 4.6. Genomic Profiling Analysis

Genomic profiling allows comprehensive detection of genomic copy number alterations. Genomic DNA of BCP-ALL cell lines was prepared by the Qiagen Gentra Puregene Kit (Qiagen). The procedure of labeling, hybridization and scanning of Cytoscan HD arrays was performed by the Genome Analytics Facility located at the Helmholtz Centre for Infection Research (Braunschweig, Germany), according to the manufacturer′s protocols (Affymetrix, High Wycombe, UK). The associated Chromosome Analysis Suite software version 3.1.0.15 (Affymetrix) was used to generate and illustrate the data.

## Data Availability

Data available on request from the corresponding author.

## Supplementary Materials

The following are available online at https://www.mdpi.com/article/10.3390/ijms231911874/s1.
