# Supplementary material for: Normal and Aberrant TALE-Class Homeobox Gene Activities in Pro-B-Cells and B-Cell Precursor Acute Lymphoblastic Leukemia
Source: Int J Mol Sci. 2022 Oct 6;23(19):11874. doi: 10.3390/ijms231911874 (PMC9570312; doi:10.3390/ijms231911874)
Supplement: Supplementary file 1 [file ijms-23-11874-s001.zip › ijms-1942385-supplementary.pdf]

Figure S1

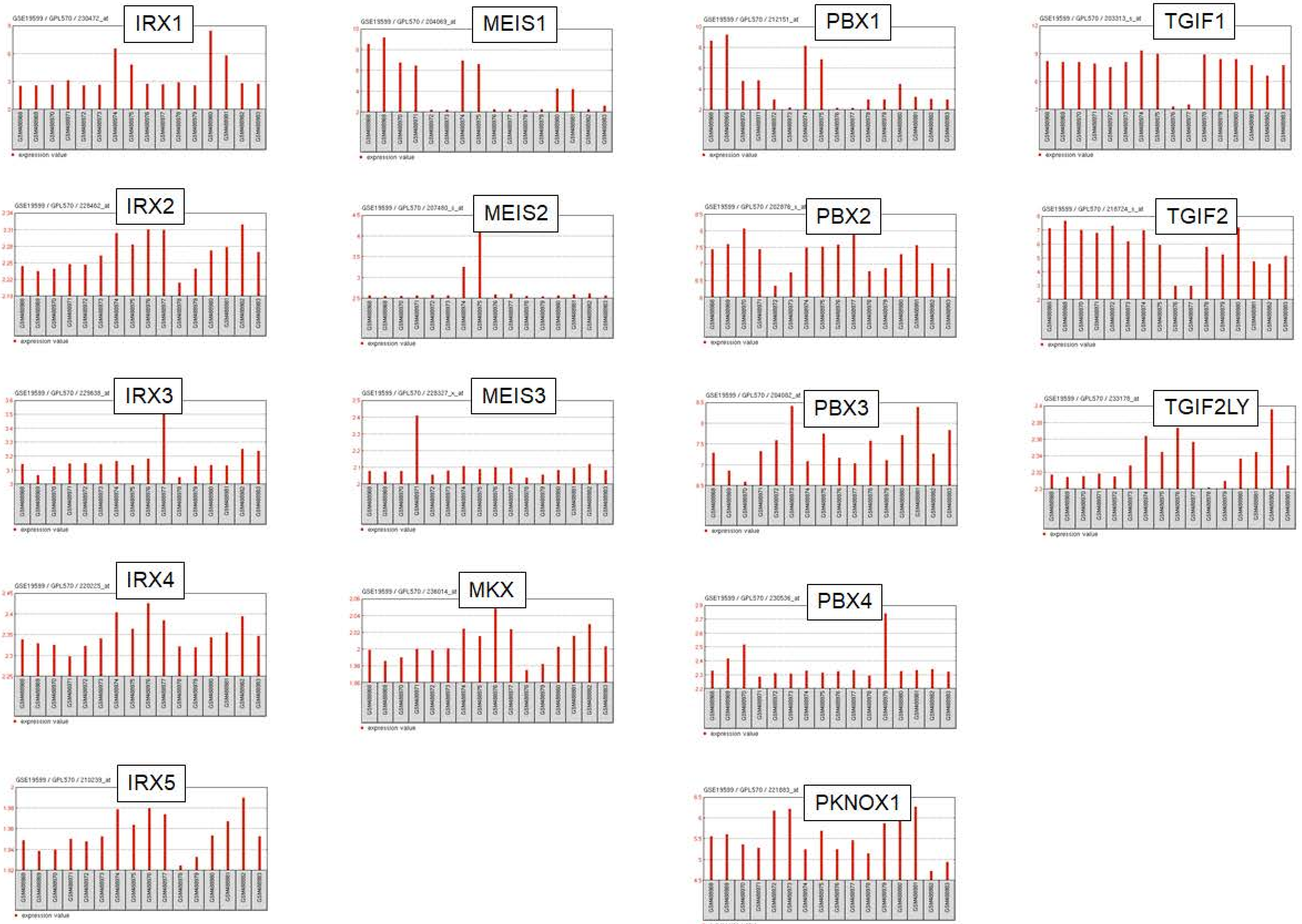

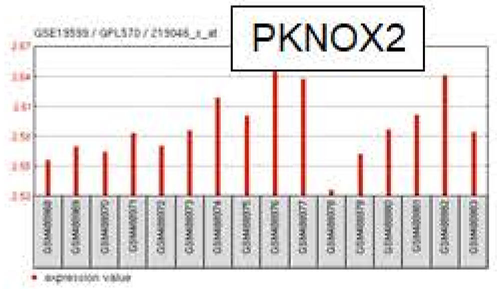

GSM488968	Flow sorted normal CMP cells, 1	Normal CMP cells
GSM488969	Flow sorted normal CMP cells, 2	Normal CMP cells
GSM488970	Flow sorted normal GMP cells, 1	Normal GMP cells
GSM488971	Flow sorted normal GMP cells, 2	Normal GMP cells
GSM488972	Flow sorted normal immature B-cells, 1	Normal immature B-cells
GSM488973	Flow sorted normal immature B-cells, 2	Normal immature B-cells
GSM488974	Flow sorted normal MEP cells, 1	Normal MEP cells
GSM488975	Flow sorted normal MEP cells, 2	Normal MEP cells
GSM488976	Flow sorted normal Myelocytes, 1	Normal Myelocytes
GSM488977	Flow sorted normal Myelocytes, 2	Normal Myelocytes
GSM488978	Flow sorted normal Pre-B cells, 1	Normal Pre-B cells
GSM488979	Flow sorted normal Pre-B cells, 2	Normal Pre-B cells
GSM488980	Flow sorted normal Pro-B cells, 1	Normal Pro-B cells
GSM488981	Flow sorted normal Pro-B cells, 2	Normal Pro-B cells
GSM488982	Flow sorted normal Pro-Myelocytes, 1	Normal Pro-Myelocytes
GSM488983	Flow sorted normal Pro-Myelocytes, 2	Normal Pro-Myelocytes

Analysis of TALE-class homeobox genes using expression profiling dataset GSE19599 which contains normal hematopoietic entities, including common myeloid progenitor (CMP), granulocytic monocytic progenitor (GMP), immature B-cells, megakaryocyte erythroid progenitor (MEP), myelocytes, pre-B-cells, pro-B-cells, and pro-myelocytes. To discriminate positive and negative expression levels we set a cutoff at 4.

Figure S2

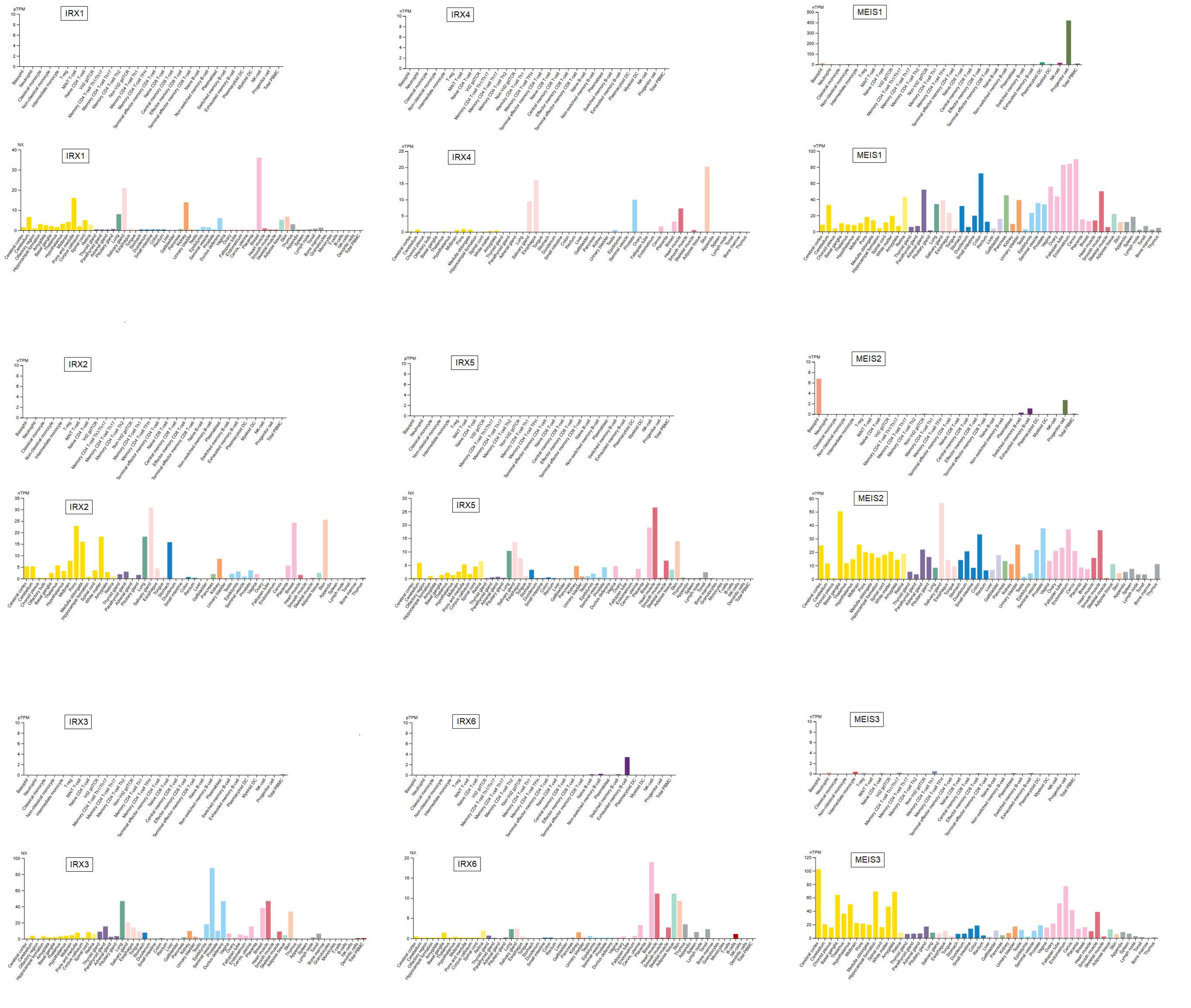

RNA-seq expression data for TALE-class homeobox genes (six IRX and three MEIS genes) obtained from the Human Protein Atlas.

Figure S3

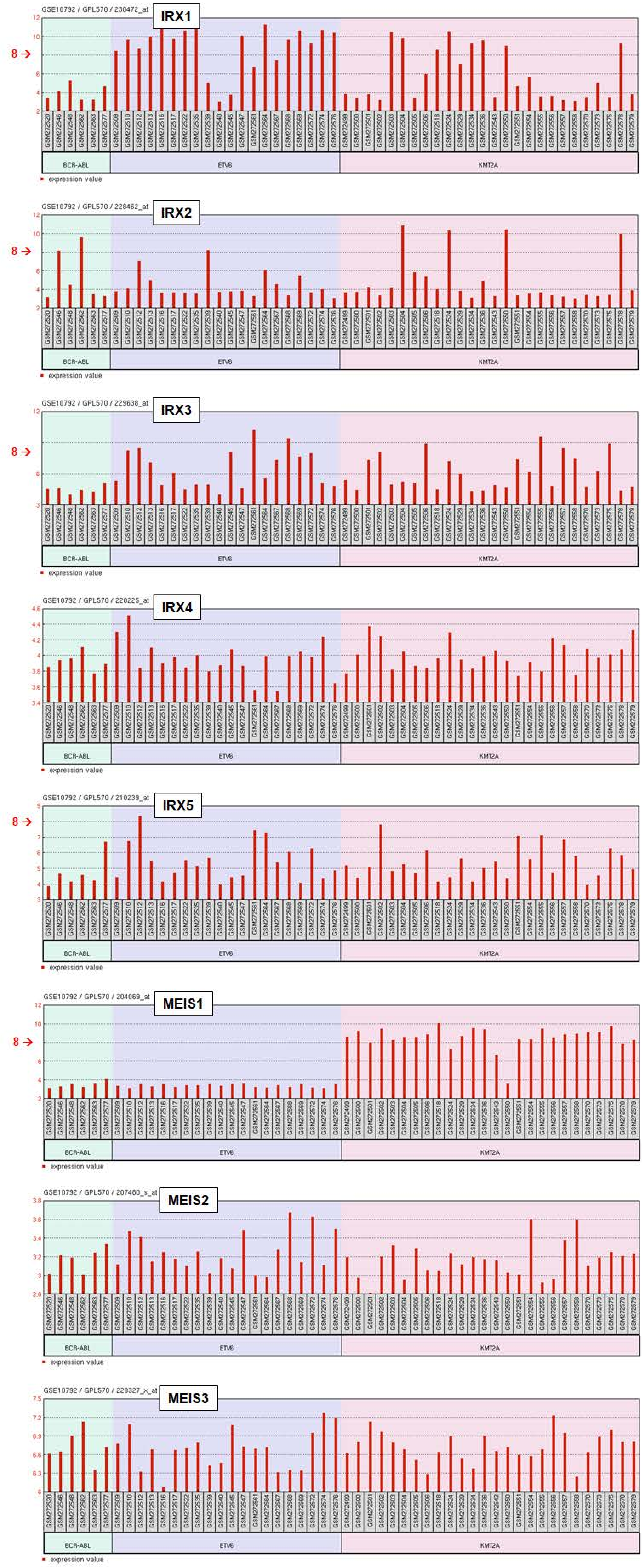

Expression profiling analysis of BCP-ALL patients using dataset GSE10792. This dataset contains BCP-ALL subtypes BCR-ABL, ETV6 and BCR-ABL. A cutoff was set at 8.

Figure S4

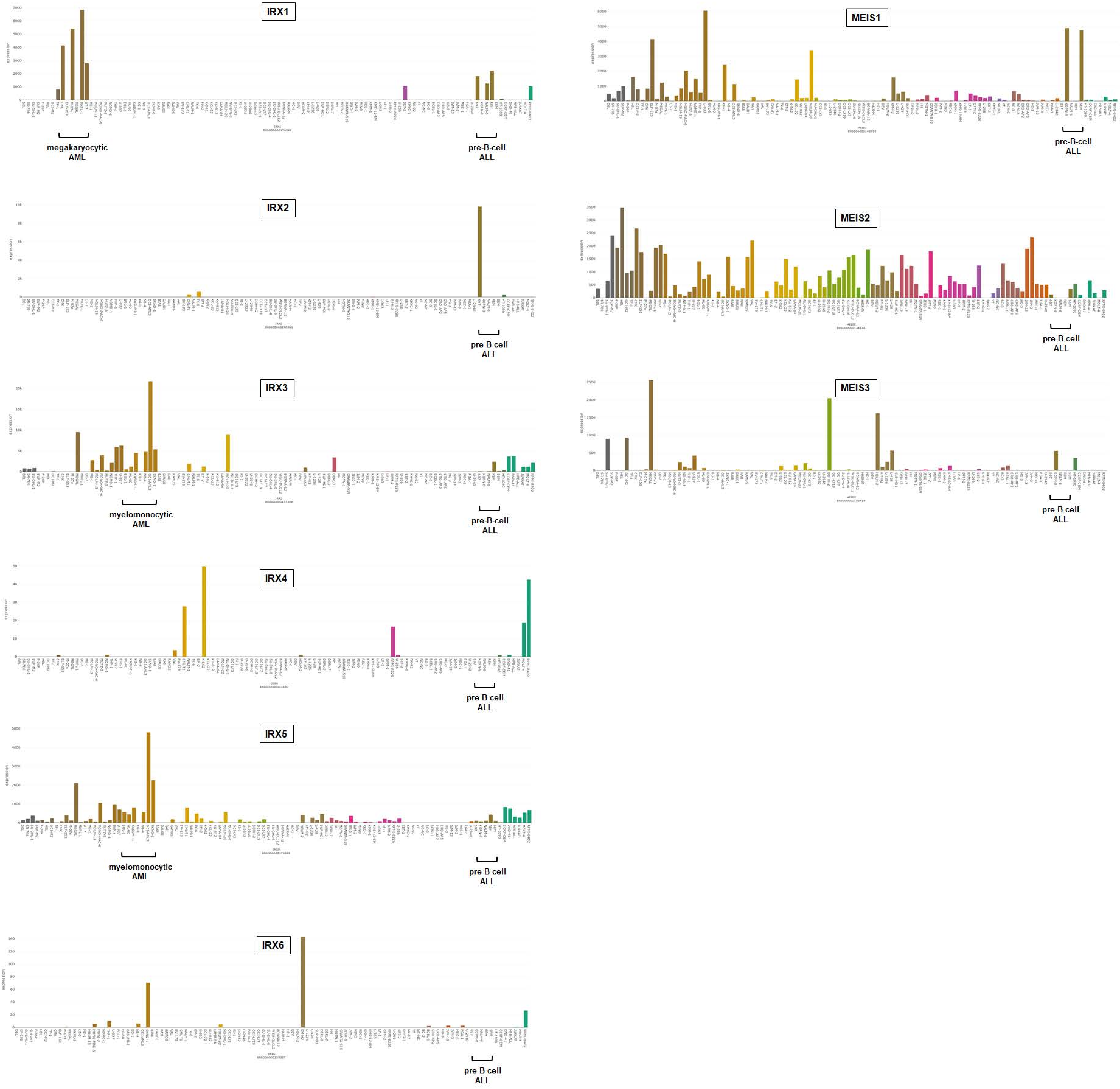

RNA-seq gene expression data for six IRX and three MEIS genes from 100 leukemia/lymphoma cell lines using dataset LL-100.

Figure S5

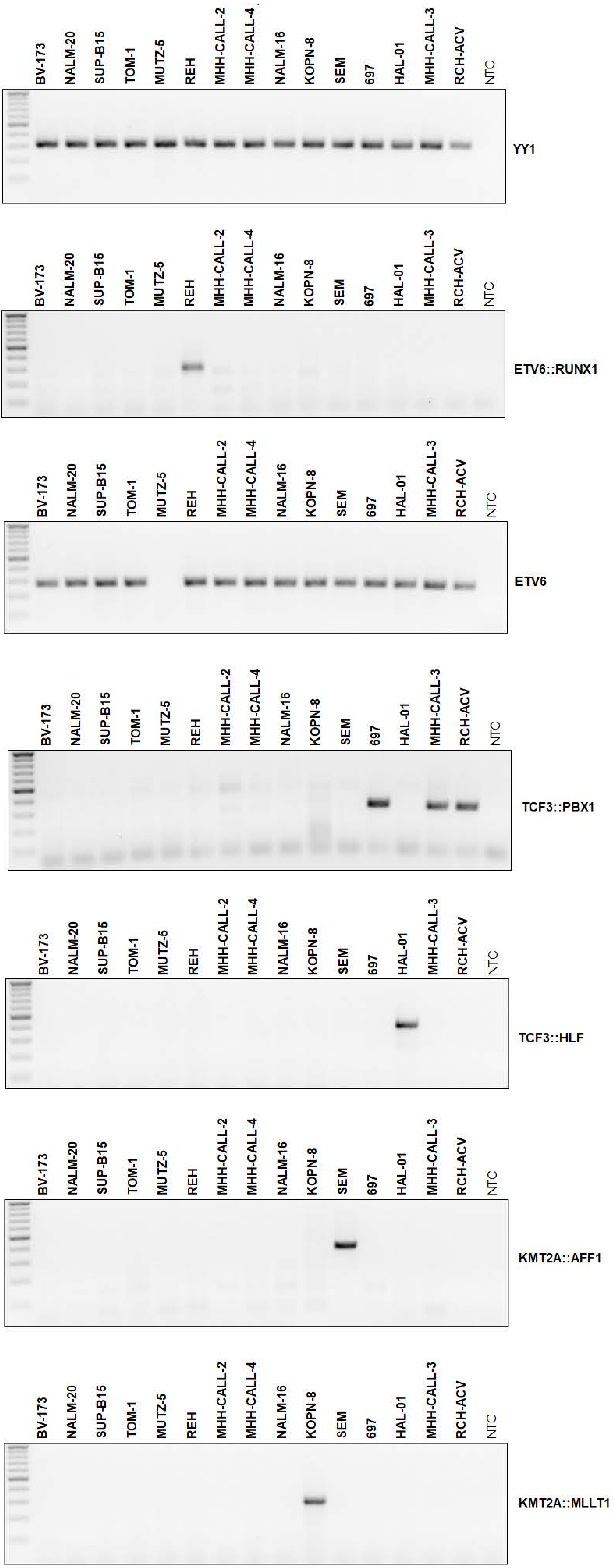

RT-PCR analysis of 15 BCP-ALL cell lines for selected fusion transcripts and ETV6.
The gene YY1 served as control. NTC: no template control.

Figure S6

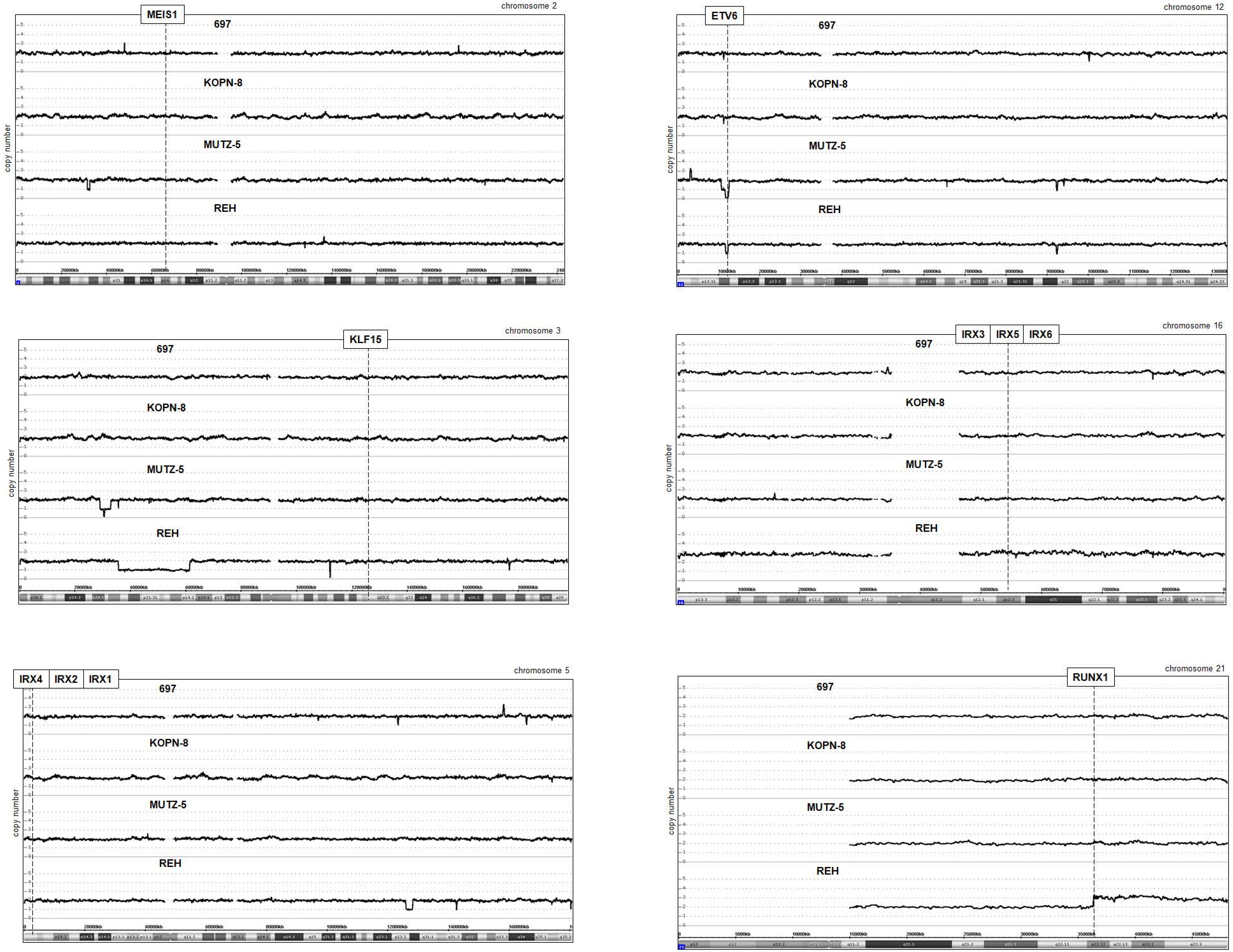

Genomic profiling data from four BCP-ALL cell lines (697, KOPN-8, MUTZ-5, REH) for chromosomes 2, 3, 5, 12, 16 and 21. The positions of selected genes are indicated. Note the homozygous and heterozygous deletions of ETV6 in cell lines MUTZ-5 and REH, respectively.
